# Quality of Mother–child Dialogue About Emotional Events, Coping and Posttraumatic Stress Symptoms Among Children Exposed to Interpersonal Trauma

**DOI:** 10.1007/s40653-021-00381-x

**Published:** 2021-07-28

**Authors:** Mathilde M. Overbeek, Nina Koren-Karie, J. Clasien de Schipper, Ivanka van Delft, Carlo Schuengel

**Affiliations:** 1 Section of Clinical Child and Family Studies, Vrije Universiteit Amsterdam, Amsterdam, The Netherlands; 2 Amsterdam Public Health Research Institute, Amsterdam, The Netherlands; 3 School of Social Work, University of Haifa, Haifa, Israel

**Keywords:** Parent–child communication, Emotion dialogue, Emotion socialization, Trauma, Sexual abuse, Intrafamilial violence, Mother–child interaction, Coping

## Abstract

Children exposed to traumatic events are at increased risk for developing symptoms of a Post-Traumatic Stress Disorder. Children often discuss emotional, and therefore also traumatic, events in their lives with their parents, and the quality of these discussions can facilitate coping and further development. The study aim was 1) to explore whether the association between the quality of dialogue between mothers and children about emotional events and children’s posttraumatic stress symptoms (PTSS) might be indirectly linked through children’s adaptive coping skills, and 2) whether this association differed when discussing different negative emotions. 169 mother–child dyads with interpersonal trauma-exposure (86% domestic violence, 14% mother and/or child sexually abused) participated in the Autobiographical Emotional Events Dialogue (AEED). Quality of mother–child emotion dialogue, captured in maternal sensitive guidance and child cooperation, and approach-oriented coping were coded from transcripts. PTSS was measured with the Child Behavior Checklist. Lower quality of mother–child emotion dialogue was associated with less approach-oriented coping and more symptoms of posttraumatic stress. There was an indirect effect of approach-oriented coping with angry feelings linking quality of mother–child emotion dialogue and child PTSS. Children’s symptoms of posttraumatic stress were reflected in the quality of mother–child dialogues about traumatic and other emotional events. Findings support that dialogues about emotional events may be a promising target for intervention with children exposed to trauma.

## Introduction

Children exposed to interpersonal traumatic events, such as sexual abuse or domestic violence, are at increased risk for developing symptoms of a Post-Traumatic Stress Disorder (PTSD) (Ackerman et al., 1998; Evans et al., 2008). Symptoms of posttraumatic stress can be debilitating and have repercussions for social and educational functioning (Trickey et al., 2012). However, not all children exhibit trauma symptoms or develop a posttraumatic stress disorder (Ackerman et al., 1998). Parental support and family functioning, as well as children’s coping styles affect their functioning in the face of adversity (Trickey et al., 2012).

After experiencing a trauma, parents are often children’s main support figure (Scheeringa & Zeanah, 2001). Children discuss emotional events in their lives with their parents, and the quality of these discussions can facilitate coping and further development (Oppenheim, 2006). The main aim of the current study is to explore how the quality of dialogue between trauma-exposed mothers and children about emotional events is associated with children’s posttraumatic stress symptoms (PTSS), and whether children’s adaptive coping skills be an indirect link.

**Fig. 1:**
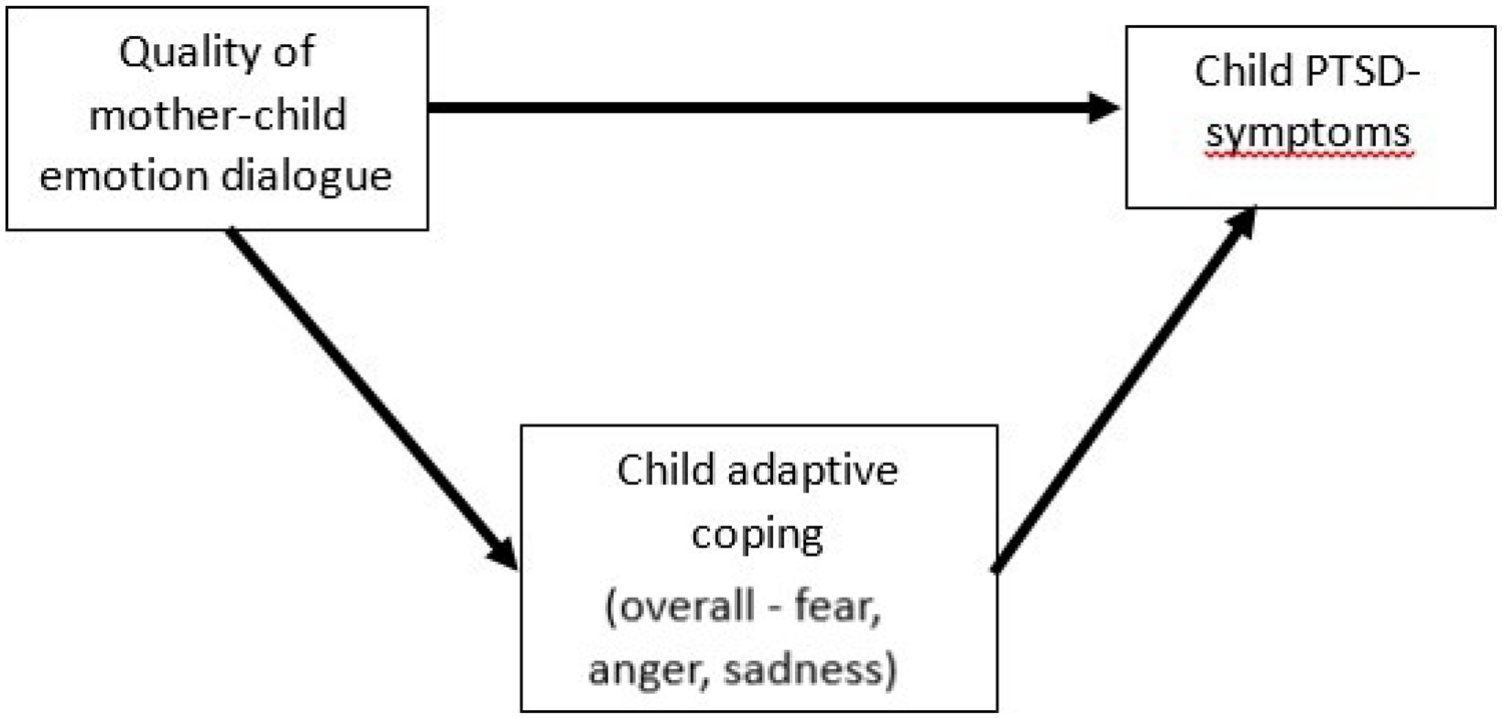
Visual depiction of the aims of the current study: explore an indirect effect of child adaptive coping (overall and with three different negative emotions independently) in the association between quality of mother–child emotion dialogue and child PTSD-symptoms

In addition, parents and children discuss negative emotions differently depending on the type of negative emotion (Fivush et al., 2003), and these differential parental reactions to different negative emotions have been found to be associated with differences in children’s functioning (Eisenberg et al., 1999). Very few studies have looked into the associations between parental reactions to different negative emotions, children’s adaptive coping and functioning. Studying differential parental reactions regarding different negative emotions in emotion conversations independently provides additional information on these individual pathways between quality of mother–child emotion conversations, adaptive coping and child functioning which may be obscured when grouping all negative emotions together. A sub aim of the current study is therefore to explore how parental reactions to different negative emotions (fear, anger, sadness) are associated with children’s adaptive coping skills and posttraumatic stress symptoms. See Fig. 1 for a visual depiction of the aims of the study.

### Trauma, Quality of Interaction and Posttraumatic Stress

Quality of parent–child interaction is reflected in communication patterns and mutual reactions between children and their parents. Sensitive parents are aware of the needs of their child and provide support when needed. Children cooperate and the interaction unfolds smoothly (Ainsworth et al., 1974). Higher levels of parental support have been found to be associated with better child adjustment and fewer symptoms of PTSD (e.g. Bokszczanin, 2008), while hostile and coercive parent–child interactions with little parental support were found to predict children’s posttraumatic stress symptoms after exposure to a diverse range of traumatic experiences, including sexual abuse and intrafamilial violence (Valentino et al., 2010). However, in families exposed to interpersonal trauma the parent–child interaction is often of lower quality than in the general population (e.g. Visser et al., 2016).

Emotion dialogues provide insight into the quality of parent–child interaction as it specifically exposes the communication patterns between parent and child. In trauma-exposed families discussion of emotional experiences is often difficult and stressful, perhaps because discussing these emotional and often traumatic experiences triggers overwhelming and negatively-loaded memories; mothers who have been exposed to marital violence showed less sensitive guidance and their children were less cooperative in mother–child emotion dialogues (Visser et al., 2016). Sexual abuse of either the mother or the child has also been associated with less maternal sensitivity and less child cooperation in emotion dialogues (Koren-Karie et al., 2008; van Delft et al., 2018).

### Coping and Posttraumatic Stress

Coping influences how an individual adapts to stressful and traumatic experiences. Coping can either be approach-oriented or avoidance-oriented. Approach-oriented coping is focused on the stressor itself and is generally seen as more adaptive; examples of approach-oriented coping strategies are seeking support or changing the situation. Avoidance-oriented coping may relieve distress temporarily, but creates distress in the long term and is conceptualized as maladaptive. Examples of avoidance-oriented coping strategies are thought suppression or social withdrawal (Littleton et al., 2007). Avoidance-oriented coping has been associated with more symptoms of posttraumatic stress in children after exposure to trauma (Aaron et al., 1999; Stallard & Smith, 2007).

### Quality of Emotion Dialogue and Coping After Trauma Exposure

Children learn how to deal adequately with daily negative emotions in conversations about emotions with their parents (Gentzler et al., 2005). In open emotion dialogues parents can offer suggestions for effective coping and at the same time serve as a role model on how to deal with negative emotions. In addition, when parents are accepting of their child’s negative emotions their children are likely to seek their support, which in itself is an effective coping strategy, as well as use other adaptive approach-oriented coping strategies (Valiente et al., 2004). Trauma exposure compromises the quality of mother–child emotion dialogue (e.g. Visser et al., 2016) and may therefore limit the possibility to develop adaptive coping skills, which in turn may be associated with more symptoms of posttraumatic stress.

### Differential Discussion of Different Negative Emotions

Parents and children discuss negative emotions differently depending on the type of negative emotion. In discussions on scary or sad daily-life events, mothers and children more often focused on resolving feeling scared or sad compared to resolving feeling angry when discussing emotional events. Resolution of these negative feelings is often achieved by an action of approach-oriented coping, such as turning on a flashlight to ward of monsters in the dark when feeling scared (Fivush et al., 2003). However, when talking about truly scary events, such as a serious injury, this focus on resolution or coping was not found (Sales et al., 2003). Also, parental differential reactions to different negative emotions have been found to be associated with differences in children’s functioning; parents responded more negatively to angry feelings than to feelings of fear or sadness. This negative response by parents was associated with more child problem behavior (Eisenberg et al., 1999). Although unstudied, this association between worse parent–child interaction and more behavioral problems could be explained by the use of less adaptive coping strategies when exposed to angry feelings.

### The Present Study

Children learn coping skills in supportive parent–child emotion dialogues (Gentzler et al., 2005). Exposure to trauma negatively impacts maternal sensitivity and child cooperation, which both contribute to the quality of mother–child interaction (e.g. Visser et al., 2016). Negative parent–child interaction may limit the possibility to learn adaptive coping skills, which in turn may be associated with more symptoms of posttraumatic stress. We first provide descriptive statistics on the associations between the individual concepts (maternal sensitive guidance, child cooperation, children’s adaptive coping and child posttraumatic stress symptoms) as well as on the use of adaptive coping by the children. Next we aim to answer the following two research questions:

#### Research Question 1

- Is the quality of the mother–child emotion dialogue related to child posttraumatic stress symptoms through children’s approach-oriented coping in a trauma-exposed sample?

Previous studies have mostly looked at parental responses to negative feelings and coping with negative feelings in general. Exploring differential parental reactions to different negative emotions will provide insight in individual pathways which may be obscured when grouping all negative emotions together.

#### Research Question 2

- Is the quality of the mother–child emotion dialogue related to child posttraumatic stress symptoms through children’s approach-oriented coping with each of the three negative emotions (fear, anger, sadness) individually?

## Method

### Participants

Participants from a study with families with a sexually abused child (*n* = 23; van Delft et al., 2018), from a study with families exposed to interparental violence (*n* = 25; Visser et al., 2016) and from a study on an intervention for families exposed to interparental violence (*n* = 121; Overbeek et al., 2012) were combined to form a total sample of 169 trauma-exposed mother–child dyads (86% domestic violence (*n* = 146), 7% child and mother sexually abused (*n* = 12), 5% child sexually abused (*n* = 9), 1% mother sexually abused (*n* = 2)). Children’s age ranged from 6–14 (*M*_age_ = 9.3, *SD*_age_ = 1.8), 54% of the children were boys, maternal education ranged from 4–25 years (*M* = 13.6, *SD* = 4.0) with three immigrant mothers completing up to 4th grade in school.

### Procedure

As part of other studies (Overbeek et al., 2012; van Delft et al., 2018; Visser et al., 2016) participants participated in the Autobiographical Emotional Events Dialogue (AEED; Koren-Karie et al., 2000). All three studies received ethical approval before the start of the study. For the current study all transcripts were recoded for the use of approach-oriented coping, and the original scores for quality of mother–child emotion dialogue were used. Independent coders coded coping and quality of mother–child emotional dialogue.

### Measures and Variables

*Posttraumatic stress symptoms* were measured with 16 items of the Child Behavior Checklist 6–18 (Achenbach & Rescorla, 2001; Verhulst et al., 1996), which have been shown to be a valid indicator of PTSS (Milot et al., 2013). Parents rated the behavior of their child on these items on a 3-point scale (0 = *not true*, 1 = *sometimes true,* 2 = *very/often true*). Alpha for this scale was 0.87. For cross-validation PTSS-scores derived from the parent-reported CBCL were correlated with scores on the parent-reported Trauma Symptom Checklist for Young Children for the children for whom these scores were available (TSCYC; Briere, 1997; *r* = 0.46, *p* < 0.001, *n* = 154).

*Quality of mother–child emotion dialogue* was assessed with the Autobiographical Emotional Events Dialogue (AEED; Koren-Karie et al., 2000), which measures both maternal sensitive guidance as well as child cooperation. In this task mother and child are asked to jointly remember and discuss four different events in which the child felt happy, scared, angry and sad. These dialogues were transcribed verbatim and scored on seven scales for the mother and seven parallel scales for the child (shift of focus, boundary dissolution, acceptance and tolerance, hostility, involvement and reciprocity, closure and resolution of negative feelings, structuring and elaboration). Each scale was scored between 1 and 9, and a higher score represented more of the coded behavior. All maternal scales (negative scales reversed) were summed in a measure for ‘*maternal sensitive guidance*’, which reflects maternal behavior which supports an enabling, emotionally regulating and child-focused emotional climate in which children can explore their emotions freely (Oppenheim et al., 2007). All child scales were summed in a measure for ‘*child cooperation*’, illustrative of a child who is involved in constructing an emotional story, elaborative, and accepting of mother’s contributions (Oppenheim et al., 2007). Because mother and child contribute both independently and cooperatively to their mutual emotion dialogue both scales were used. Coders were trained by the second author who developed the coding system (N. Koren-Karie) and established adequate reliability (ICCs ranging from 0.76 to 0.95 for maternal sensitive guidance, and 0.65 to 0.95 for child cooperation).

*Coping* was assessed by coding the transcripts for the assessment of quality of mother–child emotion dialogue. When either mother or child mentioned in the emotion dialogues an approach-oriented way for the child to feel better after experiencing the negative emotion, adaptive coping was scored. Feeling better could be achieved by changing the situation (problem/behavioral approach) or by changing how one feels about it (emotional/cognitive approach; Littleton et al., 2007). Examples of mentioned approach-oriented coping were seeking support (e.g. talking with a parent about the situation), changing the situation (e.g. turning on the light in the dark), or thinking in a positive way (1 = *yes*, 0 = *no*, for all three negative emotions. Total score ranging from 0 to 3).

*Discussion of traumatic events* were coded in the AEED transcripts as a control variable, based on the possibility that discussion of traumatic experiences may be even more difficult than discussion of other emotional experiences. Coders summarized the discussed topic per emotion and then dichotomous scoring was employed to code whether the mother and child discussed a traumatic event for this emotion. Trauma discussion could theoretically range from 0 to 4, and was then recoded into whether or not traumatic experiences were discussed during the whole conversation about the four emotions (yes/no) (see Overbeek et al., 2019 for a more detailed description).

### Statistical Analyses

All analyses were performed with SPSS (version 26). Outliers (-3.29 < z < 3.29) for PTSS (3 cases) and maternal sensitive guidance (1 case) were winsorized to the nearest non-outlier. Maternal years of education (3 missings) were imputed by the mean to limit missing cases for analyses. In two models we looked for a significant indirect effect of approach-oriented coping in the association between maternal sensitive guidance and child PTSS and child cooperation and child PTSS as outlined by Zhao et al. (2010). This indirect effect was tested with the PROCESS Macro of Preacher and Hayes (2004) for the continuous variable of overall approach-oriented coping and the Macro of Valeri and VanderWeele (2013) for the dichotomous variable of approach-oriented coping per emotion. The significance of these indirect effects was tested using bootstrapping with 1.000 samples.

## Results

### Descriptive and Control Analyses

Correlations between the different variables are displayed in Table 1. Children who were less cooperative in the emotion dialogue with their mother showed more posttraumatic stress symptoms. More maternal sensitive guidance and better child cooperation were associated with more mentioning of approach-oriented coping. Less use of approach-oriented coping strategies when feeling angry was associated with more symptoms of posttraumatic stress (*M* = 5.86, *SD* = 5.24 vs. *M* = 3.10, *SD* = 2.75; *t*(81.78) = 4.12, *p* < 0.001); this association was not found when discussing scared or sad experiences. Forty-two percent of the children mentioned adaptive coping in the emotion dialogue. Of this 42%, most children (64.4%, *n* = 58) mentioned approach-oriented adaptive coping for only one emotion, 25.6% (*n* = 23) for two emotions and 10% (*n* = 9) for three emotions. Adaptive approach-oriented coping was most often mentioned to end a discussion on a situation in which a child felt scared (50 cases – 23.3%), and to a lesser extent in discussions on when the child felt angry (42 cases – 19.5%) or sad (39 cases – 18.1%). Mother–child dyads exposed to interparental violence (IPV) were mostly comparable with mother–child dyads exposed to sexual abuse (SA), with the exception that children exposed to sexual abuse reported more symptoms of posttraumatic stress (*M*_SA_ = 10.65, *SD*_SA_ = 7.04; *M*_IPV_ = 4.53, *SD*_IPV_ = 4.03, *t*(24.32) = -4.06, *p* < 0.001) and less overall coping (*M*_SA_ = 0.26, *SD*_SA_ = 0.54; *M*_IPV_ = 0.57, *SD*_IPV_ = 0.80, *t*(39.28) = 2.35, *p* = 0.024). These differences between the samples exposed to interparental violence and sexual abuse are likely caused by the fact that part of the dyads exposed to interparental violence had received intervention (*n* = 121). In the subgroup of dyads who had not received intervention (*n* = 48), no differences were found between dyads exposed to interparental violence or sexual abuse.

**Table. 1:** Correlations, means and standard deviations of studied variables

	Mean	*SD*	1	2	3	3.a	3.b	3.c
Quality of emotion dialogue								
1. Maternal sensitive guidance	42.27	7.03	-					
2. Child cooperation	44.47	5.87	.65^***^	-				
3. Approach-oriented coping (range 0–3)	0.53	0.80	.46^***^	.48^***^	-			
a. Approach coping **fear** (range 0–1)	0.20	0.40	.31^***^	.27^***^	.68^***^	-		
b. Approach coping **anger** (range 0–1)	0.18	0.38	.35^***^	.36^***^	.68^***^	0.24^**^	-	
c. Approach coping **sadness** (range 0–1)	0.15	0.36	.24^**^	.33^***^	.61^***^	0.12	.23^**^	-
4. Posttraumatic stress symptoms	5.37	4.99	−.06	−.16^*^	−.11	−.04	−.22^**^	.01

Correlations with dichotomous variables (coping with individual emotions) were tested with Spearman’s Rho, correlations between the other variables with Pearson’s correlations

^*^p < 0.05; ^**^p < 0.01;^***^p < 0.001

### Research Question 1

- *Is the quality of the mother–child emotion dialogue related to child posttraumatic stress symptoms through children’s approach-oriented coping in a trauma-exposed sample?*

No significant indirect effect was found of approach-oriented coping with all three negative emotions combined in the association between either maternal sensitive guidance (1,000 bootstrapped CI indirect effect [-0.10 - 0.01]), nor child cooperation (1,000 bootstrapped CI indirect effect [-0.09 - 0.05]) with child PTSD-symptoms. Analyses did not differ when controlling for intervention. Arguably, discussion of traumatic experiences may be more difficult than discussion of other emotional experiences. We therefore performed the indirect effect analysis again when controlling for discussion of traumatic experiences. However, the results remain similar (1,000 bootstrapped CI indirect effect maternal sensitive guidance: [-0.09 - 0.02]); child cooperation: [-0.08 - 0.05]).

### Research Question 2

- *Is the quality of the mother–child emotion dialogue related to child posttraumatic stress symptoms through children’s approach-oriented coping with each of the three negative emotions (fear, anger, sadness) individually?*

There was no indirect effect of approach-oriented coping with the individual negative emotions ‘fear’ and ‘sadness’ in the association between quality of mother–child emotion dialogue and PTSD-symptoms (1,000 bootstrapped CI indirect effect: maternal sensitive guidance: fear: [-0.0005 - 0.0007], sadness: [-0.0010 - 0.0010]; child cooperation: fear: [-0.0001 - 0.0013], sadness: [-0.00003 - 0.00009]). However, there was an indirect effect of approach-oriented coping with angry feelings in the association between maternal sensitive guidance (1,000 bootstrapped CI indirect effect [-0.0009 - -0.0000005]), and child cooperation (1,000 bootstrapped CI indirect effect [-0.0002 - -0.00000001]). More maternal sensitive guidance and better child cooperation was associated with more approach-oriented coping when feeling angry (respectively B = 0.17, *p* < 0.001, B = 0.22, *p* < 0.001), which in turn was associated with fewer symptoms of children’s posttraumatic stress (respectively β = -0.22, *p* = 0.008, β = -0.18, *p* = 0.034). Analyses were comparable when controlling for intervention. When controlled for trauma discussion similar effects were found (maternal sensitive guidance: fear: [-0.0002 - 0.0003], sadness: [-0.0008 - 0.0005], anger [-0.002 - -0.000003]; child cooperation: fear: [-0.00007 - 0.0004], sadness: [-0.00003 - 0.00004], anger: [-0.0003 - -0.0000001]).

## Discussion

Children often discuss emotional events in their lives with their parents, and the quality of these discussions can facilitate coping and further development (Oppenheim, 2006). In this study we explored how the quality of dialogue between trauma-exposed mothers and children about emotional events is associated with children’s posttraumatic stress symptoms (PTSS), and whether both are indirectly linked through children’s adaptive coping skills. In addition we explored the indirect effect of coping with different negative emotions individually in the association between quality of mother–child emotion dialogue and children’s symptoms of PTSD. We found no indirect effect of overall approach-oriented coping—with all three negative emotions combined – in the association between quality of mother–child emotion dialogue and child PTSD-symptoms. Looking at children’s coping with individual negative emotions showed differential patterns between emotions: more maternal sensitive guidance and better child cooperation were both associated with more approach-oriented coping when feeling angry, which in turn was associated with fewer symptoms of children’s posttraumatic stress, but this indirect effect model was not found for the other two negative emotions.

### Quality of Emotion Dialogue, Coping and Posttraumatic Stress Symptoms

In supportive parent–child conversations, children learn adaptive coping skills (Gentzler et al., 2005). Also in families who have been exposed to interpersonal trauma, children with better quality of emotion dialogue with their mother used more approach-oriented coping in dealing with all three negative emotions. For families who have been exposed to interpersonal traumatic events it is generally more difficult to discuss emotional events in a sensitive way than in non-trauma exposed families. In most instances both parent and child were affected by the trauma. Potentially unprocessed and dysregulated emotions in mothers may detract from being a safe base for children from which to explore their own emotions. Mothers with significant trauma histories were found more likely to be personally distressed in response to their children’s negative emotions (Martin et al., 2018a). In addition, youth disclosed less substantive details about emotionally distressing experiences to their mothers when mothers were more personally distressed in response to children’s negative emotions (Martin et al., 2018b). This is a risk factor for child development, as we found that less child cooperation in the emotion dialogues was associated with more symptoms of posttraumatic stress in children.

Less opportunity for children to share their emotional experiences hinders the development of adaptive coping skills (Valiente et al., 2004), leading to lower resilience against posttraumatic stress after trauma exposure (Aaron et al., 1999; Stallard & Smith, 2007). The current findings additionally showed that more approach-oriented adaptive coping in dealing with feeling angry was associated with fewer symptoms of posttraumatic stress. This is – inversely—in line with the study of Eisenberg et al. (1999), which found that particularly negative parental reactions to angry feelings were associated with child problem behavior. Negative emotions such as fear and sadness expressed in internalizing behavior may lead to positive, sensitive parental responses and child recovery in a straightforward fashion, while negative emotions mixed with anger and expressed in externalizing behavior may elicit parental aversive responses (Buss & Goldsmith, 1998; Eisenberg et al., 1999). Our findings highlighted a pathway in which more maternal sensitive guidance and better child cooperation were both associated with more approach-oriented coping when feeling angry, which in turn was associated with fewer symptoms of children’s posttraumatic stress. These indirect effects models were not found for dealing with scared or sad feelings, even though both anger and fear are explicitly associated with PTSD in children (Saigh et al., 2007).

### Strengths and Limitations

In this study different samples from previous studies were combined to form a large cohort of families exposed to interpersonal trauma. Another strength is the use of an observational measure to code the quality of mother–child emotion dialogue, i.e. maternal sensitive guidance and child cooperation. Despite these strengths, our study has some limitations too, which may limit the conclusions which can be drawn from the results. We tested an indirect effect model in a cross-sectional study, so that the temporal sequence of events could not be established (Winer et al., 2016). Also, coping was not assessed with a validated instrument and therefore we could only assess whether approach-oriented coping was mentioned to deal with negative emotions in the emotion dialogue task, but not *how* adaptive the used coping strategy was. However, questionnaires have the problem that some items used to operationalize coping are confounded with distress and coping outcome (Littleton et al., 2007). Observation therefore also has some advantages and the finding that children show more symptoms of posttraumatic stress when they use less approach-oriented coping strategies when dealing with feeling angry suggests that scored approach-oriented coping strategies were at least somewhat adaptive.

### Clinical Implications

In the current sample, children’s symptoms of posttraumatic stress were reflected in the quality of mother–child dialogues about emotional events. Improving the mother–child relationship, particularly coaching on how to discuss emotions (e.g. Valentino et al., 2013), and teaching children adaptive coping skills may be a promising target for intervention with children exposed to trauma.
